# Paediatric biobanking for health: The ethical, legal, and societal landscape

**DOI:** 10.3389/fpubh.2022.917615

**Published:** 2022-09-27

**Authors:** Sara Casati, Bridget Ellul, Michaela Th. Mayrhofer, Marialuisa Lavitrano, Elodie Caboux, Zisis Kozlakidis

**Affiliations:** ^1^ELSI Services & Research Unit, BBMRI-ERIC, Graz, Austria; ^2^Centre for Molecular Medicine & Biobanking, University of Malta, Msida, Malta; ^3^Department of Medicine and Surgery, University Milano-Biccoca, Milano, Italy; ^4^Laboratory Services and Biobank, International Agency for Research on Cancer, IARC, WHO, Lyon, France

**Keywords:** children, paediatric biobanking, vulnerability, assent, maturity, engagement, empowerment

## Abstract

Biobanks play a central role in pediatric translational research, which deals primarily with genetic data from sample-based research. However, participation of children in biobanking has received only limited attention in the literature, even though research in general and in clinical trials in particular have a long history in involving minors. So, we resolved to explore specific challenging ethical, legal, and societal issues (ELSI) in the current pediatric biobanking landscape to propose a way forward for biobanking with children as partners in research. Methodologically, we first established the accessibility and utilization of pediatric biobanks, mainly in Europe. This was supported by a literature review related to children's participation, taking into account not only academic papers but also relevant guidelines and best-practices. Our findings are discussed under five themes: general vulnerability; ethical issues—balancing risks and benefits, right to an open future, return of results including secondary findings; legal issues—capacity and legal majority; societal issues—public awareness and empowerment; and responsible research with children. Ultimately, we observed an on-going shift from the parents'/guardians' consent being a sine-qua-non condition to the positive minor's agreement: confirming that the minor is the participant, not the parent(s)/guardian(s). This ethical rethinking is paving the way toward age-appropriate, dynamic and participatory models of involving minors in decision-making. However, we identified a requirement for dynamic tools to assess maturity, a lack of co-produced engagement tools and paucity of shared best practices. We highlight the need to provide empowerment and capability settings to support researchers and biobankers, and back this with practical examples. In conclusion, equipping children and adults with appropriate tools, and ensuring children's participation is at the forefront of responsible pediatric biobanking, is an ethical obligation, and a cornerstone for research integrity.

## Introduction

Biobank infrastructures play a central role in translational research (1, 2) providing a source of good quality biological samples and associated health data from a large population. Moreover, biobanks function through a network of researchers, clinicians, regulatory stakeholders and public advocacy groups, which fosters a strong platform for scientific collaboration (3). This is even more important for pediatric research, where close co-operation between multiple biobanks presents shared avenues for maximizing scarce biological samples, necessary to promote the translation of scientific advances to the development of clinical care and health policies specific to the pediatric population (4).

A pediatric biobank or a biobank with a pediatric focus, for instance, would collect samples from children with specific pediatric conditions, such as congenital disorders and rare diseases, and would follow up the children over a number of years until adulthood, thus amassing a wealth of developmental, genotypic and phenotypic data and enabling large cohort studies (5).

However, though many research institutions are committed to collecting samples from children, ranging from neonates to adolescents, there are few exclusively pediatric biobanks. Through a survey and expert interviews, back in 2010, the European Commission's Joint Research Centre (JRC) compiled a report that provided an overview regarding technical and governance issues as well as ethical aspects of, in total, 126 biobanks from 23 countries (6). It used the Organisation for Economic Co-operation and Development (OECD) definition of biobanks: “structured resources that can be used for the purpose of genetic research and which include: (a) human biological materials and/or information generated from the analysis of the same; and (b) extensive associated information” (7). The JRC report mentioned cord blood banks as well as collections of neonatal samples but did not identify any pediatric-specific biobanks.

Now, 10 years ahead, our concept of biobanks has changed, and we note a clear trend to acknowledge the border between therapeutic and research-based banks. However, therapeutic cord blood banks can dispose of units for research provided they have defined criteria for release for research, according to NetCord-FACT International Cord Blood Standards^1^. US legislation enacted in 2005, *Stem Cell Therapeutic and Research Act* (8), enables the use of cord blood stem cells in research, and at least 17 US banks are listed by the Health Resources and Services Administration (HRSA) as available for researchers. Although accredited European cord blood banks are listed in international databases such as FACT, Association for the Advancement of Blood and Biotherapies (AABB) (9) and the World Marrow Donor Association (WMDA) (10), it is not evident which of these can supply samples for research out with the transplantation field. Even less information is available about neonatal or new-born samples available from screening programmes, because these are far from harmonized in Europe. A recent survey targeting European members of the International Society for Neonatal Screening (11), revealed a significant expansion in the panel of conditions screened for since the previous survey in 2010 (12) but there is still a wide range, from no established national screening in Albania to programmes for over 40 clinical conditions in Italy (13). In fact, in Italy, the Law no. 167/ 2016 (14) provides for the inclusion of the so-called Newborn Extended Screening in the new Essential Levels of Care (*Livelli Essenziali di Assistenza*) so that free screening can be offered to all new-borns, but there is no consent foreseen for sample collection; parents/guardians can only opt out of the screening programme.

A scoping review of publications from 1973 to 2017 on research using dried blood spots from new-born screening programmes, revealed that 55% of studies originated outside the United States, with 7.4, 7.2, 6.1, 5.5, and 5.0%, respectively from the United Kingdom, Denmark, Italy, Germany, and Sweden (15). An estimated 37.5% of samples from the Danish New-born Screening Biobank, established in 1982, have appeared in publications of secondary research, with the first paper being published in 1991 (16).

A pilot study, published in 2012, targeting researchers from European biobanks, including samples from both adults and children, only identified 14 researchers working with a pediatric population (17) but did not specify how many pediatric-specific biobanks had responded. This compares with the 2012 first national biobank US survey, where only 2% of biobanks were solely pediatric banks but 44% of biobanks also included samples from pediatric patients (18). Even today, EuroBioBank (19), a network of 25 rare disease biobanks in 11 countries, whose catalog incorporates over 150,000 biological samples, gives no indication of the number of pediatric samples and datasets available for research.

Notably, the number of biobanks has increased over the last two decades, following substantial public investment into precision medicine research (20). This growth is also reflected in the Directory/Negotiator of the Biobanking and BioMolecular resources Research Infrastructure-European Research Infrastructure Consortium (BBMRI-ERIC), which now lists over 660 biobanks from 25 European countries, International Organizations and networks (21). The Directory categorizes collection types, such as birth cohort, disease specific, rare disease, population based or image collections as well as including non-human samples. It further allows a specific search by collaboration type (commercial, non-commercial use), by biobank network (e.g., Telethon Network of Genetic Biobanks), by collection network (e.g., European, Middle Eastern & African Society for Biopreservation and Biobanking, ESBB), and by data types (e.g., survey data or antibodies titer). To date, it does not allow a public search by age group. Rather, the search for “paediatric” or similar term does currently only yield results when the keyword “paediatric” is part of the name, acronym or ID of the biobank such as the King's Paediatric Liver Tissue Biobank of the UK's King's College Hospital NHS. In addition, the term “paediatric” can also not yet be selected under “collection types” in contrast to “birth cohort” or “twin-study.” Furthermore, we could not easily identify population-based biobanks with a pediatric focus, which are necessary to cover, for instance, different ethnic groups, especially when accompanied by phenotypic data (22). They also provide much needed samples from healthy children of different age groups, essential for use as controls. A good example could be a biobank of samples from new-born screening programmes.

The BBMRI community is aware of these current limitations, and is committed to tackle them, especially as BBMRI-ERIC, as a pan-European research infrastructure across more than 20 Member States, provides the ideal platform for solutions. Ultimately, these findings allow us to speculate that there is still a limited awareness about the specifics of pediatric biobanking, even among research communities. Or, one can also hypothesize that pediatric biobanks are a specificity only in some countries, such as Italy. In Italy, for instance, there are more than 50 hospitals of translational medicine with specific focus areas, including a dedicated pediatric network (23). Furthermore, there are many biobanks that are dedicated pediatric biobanks or contain dedicated pediatric collections. It is thus common to speak of pediatric biobanks or to have pediatrics in the biobank's name [e.g., *Biobanca Oncologica Pediatrica* (24)*, Biobanca di Ricerca del Bambino Gesù* (25), *Centro di risorse biologiche pediatriche – Istituto Giannina Gaslini IRCCS* (26), etc.]. Here, the pediatric biobank is an integral part of the medical system, so much so that the National Bioethics Committee published an Opinion on it in 2014 (27). Although comparisons always fall short, this particular set-up is specific to Italy and might explain why “pediatric biobank” or similar has not yet been established as a category in European catalogs.

Pediatric biobanking is challenging us above all in an Ethical-Legal-Societal horizon with its inescapable shift regarding the child's engagement as the participant throughout time. This entails a dynamic rethinking of the assenting process and the practical acknowledgment of the complexity at stake; as the minor acquires the capacity and space to participate, assenting takes on all the requirements of genuine ethical consent. However, legally speaking the parents/guardians must continue to give authorization/permission until the minor reaches the age of majority.

Such an ethical-legal shift impacts also the language. Throughout the text, we use the term child when discussing pediatric research and ethical issues in research and refer to the child as a minor when discussing legal issues related to assent and consent. We also talk about minor's assent being the minor's ethical consent, parents'/guardians' legal consent as authorization/permission, and consent attained at legal majority instead of reconsent.

## Methods

Having identified problems with accessibility and utilization of pediatric biobanks, we explored the challenges of biobanking with children with a specific focus on the ethical and legal aspects. Our own topical journey began with exchanges on best practices and challenges with colleagues from the BBMRI National Nodes and the BBMRI ELSI Helpdesk Network when we identified the need for compiling the existing knowledge. Ultimately, we carried out a narrative review including scientific publications, guidelines, recommendations, and legal texts relevant to pediatric biobanking. We used reasoning by analogy (28–30) as a more in-depth methodological approach for assessment of the regulatory frameworks. Reasoning by analogy is critical when a case or a matter, such as pediatric biobanking, is not expressly regulated; in such instances it enables the application of the rules provided for similar cases or similar matters.

Analogy was particularly fruitful in relation to clinical trials, where many similarities and enough differences to biobanking exist. In fact, the regulatory framework revolves primarily around clinical trial biomedical research in the strictest sense. The 2001 Directive (31) and the 2014 Regulation (32) specifically identify clinical trials “on” minors, and the implementation was further strengthened by the report from the expert group on clinical trials (33). It was only in 2006 that the Council of Europe published the first recommendation on research on biological materials of human origin (34) followed by the OECD Guidelines on Human Biobanks and Genetic Research Databases in 2009 (7).

Thus, we traced the evolution of ethical and legal issues of biobanking in relation to the child, their families and the research team using the analogy to biomedical research. Although guidelines are part of a body of soft law, legally non-binding recommendations, they nevertheless often carry the weight of a wide consensus of actors involved and/or field experts and may even lead to formulation of national legislation. For example, soft law relevant to basic ethical practice of pediatric biobanking includes UNESCO's International Declaration on Human Genetic Data (35), and the Universal Declaration on Bioethics and Human Rights (36). Lastly, we exemplified with cases from various countries the plethora of engagement practices or practice guidelines. In more detail, we consulted the following selected sources:

a. The BBMRI-ERIC Knowledge Base (37), which is an open-access resource platform containing information on ELSI-related matters relevant for biobanking including templates, guidelines, recommendations and legal texts;
b. A systematic-like, “narrative” literature search starting with PubMed and enlarged by snowballing methodology to identify which issues specific to pediatric biobanking have been addressed or are deemed unresolved, including specific practice guidelines by European and international regulatory bodies and influential global organizations, such as the World Health Organization (WHO) and the Council for International Organizations of Medical Sciences (CIOMS). There was no restriction on date range, and all types of articles were included. There were no exclusion criteria. Searching in the databases was made using the following MeSH keywords or free-text terms: [ELSI issues research with children], [Paediatric biobank/Paediatric bio banking], [Children AND clinical trial AND emerging issues], [Gillick competence], [Biobanking with children/^*^Bio banking with children], [Ethical legal issues AND paediatric research], [Assent in paediatric research], [Therapeutic misconception], [Right to open future], [Emerging issues research with children], [Returning result], [Informed consent AND paediatric research], [Paediatric research AND ethical issues], [Reuse of data], [Ethical issues research with children], [Research with minor] and [Research with children]; the American spelling “pediatric” was also searched for all items above (see Table 1);
c. Review of the literature (PubMed and Web of Science) in relation to the development of key ethical and legal issues in pediatric research, backed by best practices in selected institutions, identifying turning points, for which a set of recommendations for improving the ELSI framework was formulated; and
d. Selected national cases of engagement activities [e.g., in Italy, pediatric biobanking is periodically addressed on a national level through a participatory, multidisciplinary process (38) that involves all relevant players; namely parents/guardians, patient groups, caregivers, researchers, ELSI experts and biobankers].

**Table 1:** Breakdown of literature review search.

**Keywords**	**PubMed** **(July 2019)**	**PubMed** **(16 June 2021)**	**Web of science-all databases** **(16 June 2021)**
ELSI issues research with children	9	22	7
^a^Paediatric/Pediatric biobank;	^a^29	^b^30	^a^38
^b^Paediatric/Pediatric bio banking			
Children AND clinical trial AND emerging issues	36	335	289
Gillick competence	54	87	48
^c^Biobanking with children;	^c^92	^d^53	^c^101
^d^Bio banking with children			
Ethical legal issues AND paediatric/pediatric research	110	225	30
Assent in paediatric research	156	496	62
Therapeutic misconception	487	553	2403
Right to open future	502	850	12809
Emerging issues research with children	600	3998	2116
Returning result	1401	143818	299530
Informed consent and paediatric/pediatric research	1440	4538	272
Paediatric/Pediatric research and ethical issues	3135	9118	147
Reuse of data	3424	6302	27509
Ethical issues research with children	9037	18956	1742
Research with minor	117274	182497	28761
Research with children	787994	361	989
Total	925780	749092	

a Paediatric/Pediatric biobank;

b Paediatric/Pediatric bio banking;

c Biobanking with children;

d Bio banking with children.

The information gathered was independently evaluated by two members of the research team and was then grouped into five main themes:

1. A general analysis of vulnerability in children;
2. Specific ethical issues: balancing risks and benefits; right to an open future; return of results including secondary findings;
3. Specific legal issues: capacity and legal majority;
4. Specific societal issues: public awareness and empowerment; and
5. The challenge of responsible research with children.

## Vulnerability in children

Traditionally, and along the progressive regulatory journey of inclusion and recognition of the child as the participant in research (only recently does the Clinical Trials Regulation recognize the assent of the minor as pre-eminent), children have been considered by definition a vulnerable population. Thus, according to Art. 25(2) of the Universal Declaration of Human Rights, they are “entitled to special care and assistance” (39); they are subjects in evolution, in need of protection, who often cannot participate in, or take control of, the decisions affecting them. Therefore, as a vulnerable population, children require a greater level of protection, against the potential risks of participating in research, than adults.

When safeguarding a vulnerable adult, one should (40):

1. ensure they can live in safety, free from abuse and neglect;
2. empower them by encouraging them to make their own decisions and provide informed consent; and
3. prevent the risk of abuse or neglect and stop it from occurring.

In the ongoing debate about pediatric research, next to questions such as, which studies are justifiable, and for which populations of patients, it is central to define what safeguards are set up to protect children and avoid the risk of increased vulnerability. One recognizes that there may be tension between protection measures and scientific research progress. Parents/guardians, physicians, and scientists have an obligation to protect children from the harms of research but at the same time they also hope to discover new treatments for children. Thus, the conflict of interest causes tension and potentially enhances the child's vulnerability, if not appropriately and responsibly recognized and addressed early on by the adults and integrated in the research process (41).

Vulnerability and potential imbalances in power relationships may be particularly acute in research with specific groups of young people, such as children excluded from school, children outside a family structure (33) and young people with additional or complex needs, such as children with disabilities and refugees (42). Children with cognitive problems, who will not develop the capacity for discernment and choice as adults, need to be addressed separately. They will be initially supported by a guardian, their parents, or a family member, but over time by a guardian appointed by the State. This poses additional ethical and protection issues (43), particularly when biobanked samples and data could be used for genetic research. The possibility of being exposed to an increased risk of vulnerability must be considered and prevented, for example through pluralistically composed third party bodies (44) [e.g., advisory boards including citizen representative, advocacy groups, non-governmental organisations (NGOs), etc.], properly configured to represent and protect the most vulnerable.

Paradoxically, there is also the risk of overprotection in relation to categorizing children as vulnerable under all circumstances (45). This may happen if minors are excluded from research because it is considered too risky by the legal guardian(s). Consequently, the risks related to vulnerability have to be balanced with the benefits of potential research outcomes. Without translational and clinical research that is specific to children, they will ultimately suffer from the lack of development of tailored diagnosis and treatment. Therefore, overprotective measures should be avoided as they may also prove to be discriminatory.

Children are considered a vulnerable research population especially because they are not able to consent legally to their participation. They are to be distinguished from other vulnerable populations, such as individuals with mental disability or those who suffer from mental illness or other conditions that affect their capacity to appreciate the risks and benefits of giving DNA samples and phenotypic data to a biobank because their vulnerability is temporary. Their vulnerability does not arise from a disorder or predisposition. In fact, most children will become healthy adults and full members of society. Moreover, young people are expressing their wish to be listened to and engaged in decisions that affect them (46). Supporting young people in making decisions would better prepare them for the transition from adolescence to young adulthood. Consequently, the United Nations has recognized the significant and relevant participation of young people in all aspects of their personal and social development as a fundamental right (47).

A factor increasing vulnerability is therapeutic misconception. It is usually attributed to the research participant who fails to appreciate the difference between treatment and involvement in a research study or who believes that they will invariably benefit from the research. This is particularly likely with vulnerable individuals, such as children, who may experience high expectations for clinical improvement as a result of clinical trial participation. Parents, however, are also vulnerable to therapeutic misconception (48). So, it is up to the researcher or treating physician, or clinical staff to ensure that they explain the research well, in particular the likely success rate of the research. On the one hand, it might be necessary for an independent person (49) to explain the research, to avoid confusion as to the role of the clinician as the treating physician as well as the researcher, especially if the communication occurs in the clinic (50). On the other hand, communication outside the clinic may also give rise to ambiguities. It is difficult to distinguish between hope and therapeutic misconception (51) in the context of parental authorization/permission for research, more likely if the children are suffering from a life-threatening illness. In fact, a recent Dutch study revealed that the main motivator of parents to engage their child in clinical research is because it is beneficial for the child (52).

However, therapeutic misconception also occurs in researchers and in regulatory bodies. Patient organizations run the risk of portraying an overly optimistic view of the promise of biotechnological solutions, and this is often enforced via tailored communication strategies or the media to the various public(s). Advanced research involving vulnerable people implies understanding and managing uncertainty as a scientific category and acceptance of how the scientific process is set up in a probabilistic framework. Therefore, a risk assessment view needs to be enacted as a tool for capturing and managing this uncertainty to ensure active informed participation.

## Specific ethical issues

The ethical requirement for consent by research participants, to safeguard their autonomy and respect their human rights, must be distinguished from any legal requirement for consent, which is considered as an added safeguard for research participants. This distinction is problematic with respect to pediatric research since children have been considered immature to be able to give informed consent to fulfill ethical requirements while being legally considered as minors incompetent to provide valid consent. This led to the introduction of the concept of assent together with parents'/guardians' legal consent. Some however argue that from an ethical perspective the parents/guardians only give authorization/permission for their child, while still a minor. As the child grows and acquires the capacity and space to participate, assenting takes on all the requirements of genuine ethical consent. And on reaching legal majority he/she will be able to give legal consent for the first time rather than reconsent.

### Balancing risks and benefits

Research on children is underscored by the concepts of protection and best interests, enshrined in the Convention on the Rights of the Child, and guided by regulations on clinical trials, which insist on balancing benefits and risks, where risk should be minimal, that is, not higher than what a minor would encounter in everyday life.

The Principles for Good Practice for Pediatric Biobanks, endorsed by the European Society of Human Genetics (5), state that pediatric biobank research “should only be done if the research questions cannot be answered by a study of adults” and “collection and use of biological samples and data from minors should minimize physical and psychological burden.” However, biobank-based research is typically non-therapeutic and does not generate direct benefit for the participants. Rather it generates potential benefits, or potential intermediate benefits for future patients and immediate benefits for science and for society at large. It is fundamentally a voluntary participation, where the immediate benefit to the individual participant is minimal if present at all.

So potential risks cannot be directly assessed against potential benefits, especially since the risk is typically not a physical one. Indeed, biobank research is less physically risky (5), as interventional procedures are limited to those moments required for collection of the samples. Moreover, the stress due to physical intervention for blood sampling can be effectively reduced when the collection is part of the clinical routine.

Disease-biobanks can be seen as part of an advanced research therapeutic pathway involving children with specific conditions. Therefore, pediatric hospitals should ideally be directly involved in the collection of biobank samples and data during hospital admissions for clinical care. However, since the researcher is likely to be also the clinician (or the other way around), special ethical issues arise, primarily in obtaining assent from minors and authorization/permission from their parents or guardians to satisfy legal consent. Particular attention is required to avoid undue pressure on the child and/or the parents/guardians and to guard against therapeutic misconception; it is recommended to involve a separate responsible member of the research team with no strict relationship to the participant but who respects children and has experience working with them (53). We recognize the particular difficulty of obtaining assent/consent in emergency settings but in this paper, we are not addressing this specific scenario.

The types of risks related to pediatric biobanking are not just related to physical and emotional harms, but in particular for genetic information, include privacy risks associated with data processing, as well as disrespect for the child's values and opinions (43). In relation to data processing, the *General Data Protection Regulation* (GDPR) (54) attributes a prominent role to parental legal consent if the child is below 16 years of age or, provided Member States specify by national legislation, 13 years of age. Member states' provisions can be even more detailed than the GDPR and typically specific national measures complement the data subjects' rights specified in the GDPR in relation to personal sensitive data processing in health research. However, the impact for biobanking with children is yet to be determined. The literature focuses either on research or on children, and in the latter context more on issues of social media and internet vulnerability, especially toward targeted marketing. Certainly, the transfer of personal data in the context of biobank research constitutes an increased risk for children. The assessment of the risk, both of increased vulnerability and of profiling, is a critical aspect in biobanking with children, also in the light of the right to an open future. In fact, the informational risk, perhaps most critical when related to genetic profiling, arises when the sample itself has been completely used, i.e., the biological material has been consumed but the researcher-generated data continues to be accessible as part of the scientific circuit and remains technically reproducible and usable *in silico*, its usage almost unlimited by technical possibilities and only by data privacy laws such as the GDPR. By the time minors are re-contacted to express their consent as an adult, and choose whether to retain their samples in the biobank with the same conditions, their identifying DNA sequences, along with their particular data sets, may have already been shared with other resources around the world.

Furthermore, with genomic research there is a long-term risk horizon at stake. At present, “the risk of re-identification is often difficult to assess” (55) but this is a possible outcome when big data from genomic research is subjected to algorithmic analysis. Moreover, with the use of Artificial Intelligence and Machine Learning, group harm may develop due to “group-based generalizations and inferences” (56). There are therefore potential future consequences not only for health issues but also for harm from discrimination and stigmatization (55), affecting child participants and their families, as well as unknowingly, participants who suddenly find themselves in a specific group codified by the new technologies (56), with a wider impact on society. Regulatory frameworks and policies to safeguard participants are much slower to develop than advances in technology, so enhancing the susceptibility of children “to the long-term ramifications and inappropriate applications of data” (57). These future scenarios are unclear and therefore a big challenge for Institutional Review Boards (IRBs) (56) and Research Ethics Committees (RECs) (58), raising issues about their regulated mandate and expertise (56, 58).

Re-use or secondary use of samples and/or data is a characteristic feature of a research biobank, so biobank governance procedures must be in place to ensure that proper informed consent has been obtained at the collection time and/or have procedures in place to re-contact the research participants whilst respecting their autonomy. Besides satisfying ethical requirements, consent procedures need to be appropriate to fulfill the national legal obligations, especially when used as a legal basis for data processing.

The storage and use of these samples as well as of the related, particular data may violate the autonomy, privacy, or personal integrity of child participants. The distinctive physiological and emotional development “may also place children and adolescents at increased risk of being harmed in the conduct of research” (59). Children and their parents/guardians should therefore be made aware of the most likely to occur privacy risks associated with the transnational sharing of genomic data. All risks, will never be known and that also needs to be communicated. Dynamic consent procedures can be one way of allowing for adjustments as new information and risks arise and can be one of the measures used to keep research participants aware of the research progress.

Striking the right balance on risks and benefits is most critical. The debate on risks and benefits will ever be on-going. Consider for instance, a recent line of thought in medical research which stresses the fact that research with, and for children, is legitimate whilst arguing that a focus on the risks does not do justice to either the participating children nor to the children benefiting from the research. In support of this, a recent US study concluded that 55% of parents were willing to include their youngest child in biobank research, even though only 58% identified benefits to their child, while 80% acknowledged benefit was for other children (60).

### Right to an open future

Parents continuously make many important decisions that affect their children's future. In case of biobank participation, however, the outcome of the parents' choices may have unpredictable consequences decades later and should not be left to them alone.

Uncertainty about the probability of the research findings can have quite an important impact. Parents might hope for definitive answers about their child's illness. They may use the genetic research findings in making future decisions about their child's health and their own future family planning. But potential benefits are tempered when the child will not develop disease until adulthood. Imposing this knowledge can interfere with the child's “right to an open future” (61), which refers to the idea that children will become autonomous once they reach the age of majority, and therefore parents' decisions should be such as to ensure an autonomous choice when they are ready.

Moreover, children may have developed different values than their parents and may not agree with certain types of research or certain procedures, to which the parents consented without any difficulty. There is therefore a risk of ethical violation, with disrespect for the child's values and opinions (43). The debate is considerable, and most experts state that parents should not have the right to decide about the right to know or not to know on behalf of their child.

### Return of results, including secondary findings

Since pediatric biobanks often deal with genetic results, there is now focus in the literature on return of secondary genetic findings. There is a growing consensus that secondary findings that are “actionable in childhood” should be reported to the parents and “actionable” has been well-defined as follows: “if there are available preventive and/or treatment measures and the disorder has either (i) childhood onset and such measures are therefore initiated in childhood, or (ii) adult onset, but such measures have been demonstrated to be effective when started in childhood” (62). This is a welcome definition since there has been controversy about the recommendations by the American College of Medical Genetics (63) to report secondary findings from clinical exome and genome sequencing, even for adult-onset disease (64). Generally, parents believe they have a right to receive genetic results (65) and are in favor of receiving all types of results (66) from biobank-based research (by the biobank or researcher directly). If parents opt not to receive genetic results, which are considered actionable, it has been argued that it is the researcher's duty to ensure the children are protected, and in some cases even override the parents' right not to know (67).

For adult-onset diseases, only actionable in adulthood, there is less consensus (68). Some have argued that parents should decide whether to receive results or not (69). However, children's rights, autonomy and their right to an open future are best served if they are allowed to decide themselves whether to know or not, when they become adults. Since such diseases are predominantly inherited from the parents, return of genetic results has consequences not just for the child but for the entire family. It has been argued that children would benefit if results are returned to the parents, so they can remain healthy and be around for their children, even though this may be compromising the child's right not to know on becoming an adult (68). Others have shown concern when parents refuse return of results from genetic studies on their children because they might indicate a genetic disorder in the parent (70).

Moreover, according to many experts, an older child, especially a mature minor, can decide independently whether to be informed about his/her individual findings. In any case, the child should be informed about his/her samples and data and ideally, on request, be able to obtain information regarding individual findings (71).

There is widespread consensus that participants should receive relevant and pertinent information but there are no specific guidelines on its quantity and timing of delivery. It is out of the scope of our paper to go into further details, but there are many studies that show that participants, parents and the public in general are longing for tangible information (68). While others suggest that once there is more knowledge available, including genetic counseling (72), the participants learn to consider risks and are more cautious about receiving genetic results, while others talk about consent fatigue and information overflow. Again, striking the right balance is critical.

Certainly so, the biobank is, as an infrastructure, a critical actor and shares some responsibility with the researchers and clinical staff. Undoubtedly, a qualified person needs to be mandated to provide feedback to participants. Some population-based biobanks, for instance, have a genetic counselor on staff and have the legal obligation to share findings. In most countries, however, it is the responsibility of the treating physician, and the biobank might just have an intermediary role without direct contact to the participant. Whatever governance model a biobank has implemented, this process should be transparent and provided prior to obtaining assent from minors and authorization/permission from the parents/guardians for legal consent to sample and data collection. The Public Population Project in Genomics and Society (P3G) International Pediatric Platform recommends discussion about return (or not) of genetic results should occur during the informed consent process (73). Ideally participants should have an opportunity to change their preferences throughout the lifespan of their samples and data. A practical solution that is becoming available is to invest in digital tools, including online portals that facilitate communication with participants (74). If a biobank has direct contact to research participants, this might be an investment to consider.

## Specific legal issues

### Minors' capacity and legal majority

The term minor used in Article 4 of the *Clinical Trials Directive* and Article 32 of the *Clinical Trials Regulation* for children who are still to reach legal majority, is now applied to participants in all pediatric research, emphasizing the legal responsibility of parents or guardians in the process of consent. Article 32.2 highlights that “the minor shall take part in the informed consent procedure in a way adapted to his or her age and mental maturity.” Yet the Committee on Clinical Research Involving Children from the Institute of Medicine, US, argues that in fact parents and legal guardians can only give permission and not consent for the minor to take part in research because “only those who are held competent to make autonomous decisions on their own behalf can provide informed consent” (75), a viewpoint also endorsed by European ethicists (76, 77). The report further emphasizes that an investigator may exclude a child from participating in research because they are concerned that the parents are failing in protecting the child's best interests. Such a situation may be related to therapeutic misconception by the parents.

Provision of informed consent in pediatric research is tied to the constraints imposed by the national age of legal majority. A set age would help in harmonizing biobank policies but, as stated earlier, there is no uniformly set legal age across all European countries. Age limits for consent to treatment have been addressed in various ways, ranging from a fixed age for legal consent, to an age threshold provided capacity is demonstrated, to a fixed age limit with consent allowed if capacity is demonstrated at a younger age (e.g., Gillick competence), and to variable age limits with joint legally valid consent by parents and minors, related to competency such as in the Netherlands (78). The European Agency for Fundamental Rights has mapped the European landscape regarding the age of consent by minors for medical treatment without parental consent (79). However, there has been less attention to the establishment of an age of consent specifically for research, though some countries have set a legal age just for clinical trials, for example in the UK legislation, “minor” means a person under the age of 16 years (80).

Assent by minors who “are deemed legally incompetent” was introduced as an ethical requirement in the *Declaration of Helsinki* (2000) (81) and was accepted the following year in European legislation, namely, the *Clinical Trials Directive*, and now in the *Regulation*. In the Recommendations of the expert group for the implementation of the Regulation (33) assent is considered to be “a statement of will with legal value according to national law.” However, for countries where assent “is not a legal requirement” the term “agreement” is used as an analogy to assent.

Though recommendations to introduce assent and to involve the IRBs, were formulated in the US in the late seventies (82), there are still no federal regulations as to the acceptable age of assent. The IRBs are responsible for determining if assent is required, taking “into account the age, maturity, and psychological state of the children involved” and have to “determine whether and how assent must be documented” (83). Though there is no consensus on the recommended age of assent, the IRB may consider the age of 7 (75), in keeping with the recommendation of the American Academy of Pediatrics (84). The Committee on Clinical Research Involving Children from the Institute of Medicine states that children aged 6 to 10 years “can understand the more practical features of research but show a lesser appreciation than older children of the more abstract features of research (e.g., understanding of risks)” (75). By 14 to 15 years of age, adolescents are considered able to make similar decisions as adults with regard to research participation. A survey of Canadian adolescents and their parents as to the appropriate age of assent to research participation, revealed that adolescents opted for a median age of 14.5 years, while their parents favored 16 years (85).

Though Hein argues that children from the age of 12 can give consent (86), and this is the case for clinical trials in the Czech Republic and the Netherlands (87), there is still no consensus in the EU on the age of assent, as shown by the survey document (87) developed by the Working Group on Ethics at the European Network of Paediatric Research at the European Medicines Agency (EnprEMA), where countries specified varied age ranges for consent and assent in clinical trials, starting from the age of 4 years.

However, a specific legal age of majority fails to take into consideration the developing maturity of the child. In fact, such variation in accepted legal age reflects the increasing recognition of a difference in children's neural development with cognitive capacity approaching that in adults by the age of 18 years while psychosocial maturity lags behind (88, 89), resulting not only in development of different abilities with time but also making the adolescent more prone to erratic behavior under emotionally charged situations.

Therefore, it is not surprising that there is a lack of guidelines regarding the assessment of decision-making capacity for assent, specifically in research.

In formulating guidelines about consent/assent for clinical trials, the Directive refers to “capacity of understanding” of the minor, which was evolved to “age and mental maturity” in the Regulation, in line with an increasing appreciation that cognitive development, which occurs over a period of time as the child matures, determines the process of consent. In fact, the capacity to consent has been characterized as having four elements: “(1) understanding the information relevant to make a decision, (2) appreciating how the decision will impact them personally, (3) manipulating the information rationally and reasoning, and (4) communicating a voluntary choice” (90).

A recent Delphi study that consulted pediatric researchers, and regulators with expertise on ethical and legal issues pertaining to pediatric research identified the following two main methods for the researcher to assess maturity: “discussion with both the parents and child to gauge maturity/cognitive ability” and “feedback to assess understanding” (91). The use of general development, age cut-offs or a standardized tool, like the MacArthur Competence Assessment Tool for Clinical Research (MacCAT-CR) (which covers the four capacity standards) achieved much less consensus, even though Hein (92), had used it to enable a conclusion that children were competent to assent to research at 11.2 years, though this relies on an emotionally neutral environment (88).

This raises the issue of how best children may receive information about research. In Europe, both the Directive and the Regulation for clinical trials, respectively, mention the need for information being explained by “staff with experience with minors, regarding the trial” and “investigators trained or experienced in working with children” and for these researchers to consider the wishes of a minor provided the child is “capable of forming an opinion and assessing the information.” Minors' views “shall be taken into consideration on matters which concern them in accordance with their age and maturity” (93) but there are no specific guidelines available indicating the parameters for assessing the maturity of the minor and neither are there indications as to who should best assess minors.

The 2017 Recommendations of the expert group on clinical trials for the implementation of Regulation 536/2014, *Ethical considerations for clinical trials on medicinal products conducted with minors* (33), refer to documented “discussions between the investigator, the parents/legally designated representative and the minor,” to assess maturity in relation to “developmental stage, intellectual capacities (e.g., children with special needs and/or learning difficulties), and life/disease experience” (33). This naturally should apply to all types of research studies, including in relation to biobanking.

Appropriate expertise includes not only knowledge of child physiology and development but also a familiarity with pediatric clinical care and research and the accompanying ethical issues and regulatory obligations, including also the family logistics (75), which may affect the balance of decision-making, since children place different emphasis than their parents/guardians on the type of information they deem important to consider prior to decision making (94). This expertise would allow the researcher to adopt different methods in interacting with the minor, relating the approach to the minor's capacity to understand the information, bearing in mind that cognitive development depends on culture, and experience of the child. There is a paucity of research that has investigated the actual process of obtaining minor's assent and outcomes, in terms of the quality of research participation.

The concept of assent should focus beyond the provision of information and the fulfillment of ethical and legal requirements. It is the end result of “appropriate engagement” with the minor (95). The process of interacting with minors displays support for their developing autonomy, fosters communication between the researcher and the minor and “contributes to the child's upbringing and moral education” thus playing a major role in the engagement of the child in research (96). Assent procedures also enhance the view that the decision-making process is fair and promote self-confidence in the minors (97).

As children mature, their understanding of risks, benefits and ethical issues, such as their rights, increases. The question of developing maturity makes a strong argument for considering layered assent procedures, such as dynamic informational tools (98), which allow both minors and parents to be consulted on a regular basis throughout the research project, targeting the right amount of information to be shared with, and explained to, the participant. Under this perspective, parental authorization/permission and child assent are the two critical components (77), especially for children under 12 years old. Only at attaining the legal age, will the minor be asked to give consent to biobanking. Now the challenge is to fully recognize assent as a process (98) tailored dynamically together with the minor and framed with a developmental approach. The role of the minor in this process of “dynamic assent,” for instance, has been described as personalized assent, where both the informational content and the practical process of obtaining assent are adapted to an individual child's capacities and wishes (53, 96).

### Consent on attaining legal majority

Another issue that is specific to pediatric biobanks is what happens to banked specimens once a child attains legal majority. It is generally accepted that these cannot be used for further research, and associated data cannot be processed further, unless there is re-contact with the individual. In a study by Kong et al. (85) on Canadian adolescents (14–18 years) and their parents, from two equal groups, from clinic attendance and school attendance, there was almost the same response. Fifty percentage of children from the clinic and 64% of their parents and 55% of school children and 63% of their parents considered it important to be re-contacted before samples are used again. However, around half of the adolescents and the parents were also in favor of continued use of their samples if the re-contact was not possible. Opinions of young adults are in favor of reconsent on attaining majority (99), which is not surprising since in actual fact the young adult is actually consenting for the first time. Therefore, as already explained, we have avoided using the term reconsent. Biobanks should ascertain the opinions of adolescents on preferences regarding re-contact. There appears to be support for consent on attaining majority, as this recognizes their autonomy and respects their assent. Knowing adolescents' preferences can help to implement biobank policy in this matter (100). Re-contact is viewed as a way of respecting the autonomy of the young adult, but also as a “positive interaction” (101) and a means of building trust in the relationship with researchers. The long-term benefits of maintaining public trust in biomedical research by waiting for participating minors to consent as adults justify extra governance efforts and added costs (102). The use of digital means of re-contact and electronic consent should make this process more easily feasible.

## Specific societal issues

### Public awareness and engagement

Informed consent from the parents or legal guardians and assent of minors is made easier if there is awareness of the role of translational pediatric research and of biobanking. This is obvious to those working with families with rare diseases, who have access to patient advocacy groups and are generally well-informed as to possible benefits of research to their children (103) and are even proactive in pushing their children forward to participate in research. Of course, one must be careful of not falling into the trap of therapeutic misconception, as already discussed.

A practical example emerged from a study carried out at a US pediatric intensive care unit, where it is routine practice for administration to approach parents on admission, with a consent form for enrolling the child in the pediatric biobank. However, out of 80 parents that later agreed to take part in a study, only 54% confirmed that they had already been approached in relation to research (104).

Awareness in the general public about the need for biobanking, especially for effective pediatric research is very much in its infancy. This is likely a reflection of poor public awareness of the role of biobanks in all research. There is a role for using modern technologies to disseminate information, such as a website specific to a project, but there should be more effort in educating the general public in science and research in general, starting with a well-organized programme at schools to provide a science framework.

An example of a successful activity is the initiative of the Italian National Node that took place for the first time on the occasion of, and in partnership with, the European Biotech Week 2017; during the initiative “Open doors in biobanks” high school students systematically experienced a real hands-on participation at a biobank (105). As proof of its success, the initiative is now a well-established fixture at every Global Biotech Week.

Tailored tools can be developed for awareness and engagement for children appropriate to different age ranges. For secondary school children, visits to biobanks can be organized and they may be introduced to the usage of attractive digital tools (video, clips, social networks, apps, blogs, forums, etc.), which have more impact for young people.

The promotion awareness and participation can be emphasized by providing examples of success stories for biobanked samples that have contributed to successful research projects. A best practice example is the Gambian Hepatitis Intervention Study (GHIS), a large-scale vaccination project in The Gambia, initiated in July 1986, in which the introduction of national hepatitis B (HBV) vaccination of young infants progressively over a 4-year period was proposed (106). This program, based on epidemiological data that were validated by the analyses of linked biobanked biological samples, has demonstrated the efficacy and effectiveness of infant vaccination against chronic hepatitis B. The GHIS strategy provides a model for integrating and evaluating new vaccines into the Expanded Programme of Immunization of sub-Saharan African countries.

To make research easily understandable for everyone as well as to advocate research results and requirements appropriately, research needs to become an ordinary issue and engagement activities have to be conceptualized, executed and maintained as such (107). Pediatric biobanks situated within, or working with pediatric hospitals (4) are ideally placed to ensure this happens; they are in the best position to obtain the maximum number of samples and as early as possible during disease, but also to engage with children and their parents/guardians. Consider, for example, the Precision Link Biobank for Health Discovery (Boston Children's Hospital); it was created to provide valuable human samples for researchers to advance knowledge of health and disease. The biobank holds the health information and samples (such as blood, tissue and cells) of thousands of patients and their families at Boston Children's Hospital. A comic book was developed for educating children about medical research and a video overview of several aspects related to medical research is available for parents/guardians. Guidelines and policies used, including regarding education and training, research activity review, informed consent/assent, and vulnerable populations, are easily available (108) on the website. A similar approach was followed for the Born in Guangzhou Cohort study (109) at the Guangzhou Women and Children's Medical Center, where information is available for the thousands of patients and their families through a secure online infrastructure, complemented by children-specific informational material, and highly trained staff.

Today, there is a well-established role for patient advocacy groups and NGOs to become involved in events that educate their members and the general public about research involving children. This collaboration leads to a better understanding of research by participants, and a greater understanding of the needs and interests of participants by researchers. Increased awareness of medical research initiatives in the scientific community, as well as in an age-appropriate manner, make it easier for children, young people, and parents/guardians to decide to participate, if they are ever invited to do so. Equally so, policy makers have to be engaged in a dialogue.

## The challenge of responsible research with children

The process of building a relationship between the adult researcher and the child to allow participation in research has been explored in the context of qualitative research in early childhood (110) but not so much in the area of scientific translational research. This is surely an area which needs to be developed as it ensures investment for collaborative lifetime research.

Researchers have a responsibility to conduct ethical research, which includes the principle of “protection of the participants' interests” (111), particularly protecting vulnerable groups. Researchers have a central role in establishing effective communication and engagement with the child participants to ensure whether the children are able to, and actually do, understand what the research entails and what are the benefits and most importantly the risks. Only such engagement will ensure real informed and personalized assent, which takes into consideration the life circumstances (96). Giesbertz argues that once a researcher acknowledges “a child's right to personalized assent,” they have a “moral duty” to make a “best effort to engage the child” (53).

Practical suggestions of how this can occur include a decision as to which information should be provided, how best to present it, and who is the best individual to shoulder this challenging task. One should first concentrate on practical information, which surrounds the sample collection process. More abstract concepts, like privacy should be discussed later on as the child's capacity develops (53). It is important to use different types of material as well as verbal and digital means of communication.

We advocate that children's involvement should be at the front and center of pediatric biobank research, particularly in tackling the complex specific practical and ethical issues that need to be addressed to ensure that the child is personally and appropriately involved in the decision-making process. A main issue remains, who is best in a position to engage with the child. Though this may seem to be the responsibility of the parent(s)/guardian(s) or researcher, in view of the fact that the minor should not be coerced into assent, there needs to be investment in the training of competent staff to interact with minors and obtain their assent (53).

Consultative processes can be made participatory by, for example: enabling children to identify what the relevant questions are; giving children the opportunity to help develop the methodology for the research; allowing children to take on the role of researchers; involving children in discussions about the findings, their interpretation and their implications for future developments (112).

However, since for legal consent, the minor's assent also requires the authorization/permission of the parents/guardians, the researcher has also to be trained how to communicate with the parents and/or even other members of the family, who may also have a vested interest in the results of the research. One may be falsely reassured by the filling of consent forms. These are, however, too often just a measure to pre-empt litigation and cannot really replace the value of good communication and good ethical practice.

It is thus a good investment to involve the research participants' parents' or even their family in dynamic processes of engagement in biobank research. A good example to follow is the collaborative approach tried in Canada, which specifically introduced participation of parents and patients (113) at various levels of the research labeling them as a “principal knowledge user” on funding application forms and engaged them in the organization of the approach to participants and in assessing protocols (104). This is a practical and sure way of building trust and is also a protection measure, minimizing children's vulnerability.

There is a general consensus that the success of a positive outcome for engaging participants depends on the level of communication or “meaningful engagement” between researchers, parents and children. Yet, there is a dearth of literature to back this up and to provide the best practice methodology to be used.

## Training of research teams

As stated earlier, staff training is critical. Indeed, the responsibility is not to be borne by one researcher but needs to be shared by the entire research team and lived as daily practice and guided by research integrity. Biobankers are largely aware of the responsibility that the biobank has as a guardian of biospecimens and associated data in line with sound ethical practice and robust adherence to local and international legal frameworks (114). The literature mentions the need for biobank staff training, in general terms (115) but there is no specific publication on ELSI awareness training.

There are very few higher education courses educating a young generation of biobank staff (116, 117), and some are listed by the International Society for Biological and Environmental Repositories (ISBER) (118); part of these courses is dedicated to presenting the ELSI perspective in biobanking (116, 119). BBMRI-ERIC delivers webinars dealing with specific ELSI aspects in biobanking (120) and ESBB offers educational programmes, which include webinars on ELSI (121). However, there are no known, established official ELSI-themed training programmes for scientists to work in a biobank (122) or established harmonized paths by which already trained biobank staff can further train other staff and researchers outside of some training sessions in the context of research projects (e.g., the European Joint Programme on Rare Diseases) (123).

Training opportunities have been created to service *ad hoc* needs within isolated pockets of excellence. For example, the Collaborative Institutional Training Initiative (CITI) in the US offers training for researchers aimed at protecting participants in human research, and nearly all IRBs require evidence of training (124). There are training requirements for researchers at some universities (125, 126) but specific pediatric training is not identified.

There are well-documented problems with training of pediatric researchers. In the US, it is estimated that <10% of pediatric medical subspecialists devote at least half of their professional time to research (127).

A good practical example is the UK Child Health Research Collaboration, a partnership of various funding agencies supported by the Wellcome Trust and Medical Research Council, with the aim of fostering “collaborative solutions” between stakeholders involved in pediatric research. They aim to “increase research capacity,” including training and also to “strengthen research infrastructure” and “improve parent involvement and public awareness of children's research relevance and importance” (128).

Training or orientation for the engaged population was reported in a few articles. Although the regulation on pediatric clinical trials specifically mentions “investigators trained or experienced in working with children,” there appears to be a lack of training programmes specifically targeting research in pediatrics. Though programmes can follow the basics laid out for general research (129), we have tried to pinpoint that pediatric research requires additional ethical considerations. It is good to see that there is increasing awareness of this need for preparation of researchers and it is encouraging that there are national attempts to address this issue, such as the specific training programmes for clinical researchers in Europe, although few are currently mandatory (130).

## Discussion

Most notably, there is a lack of knowledge about the causes of, and appropriate strategies for prevention and care of diseases in childhood. These diseases can never be understood by looking only at adults (131). Scientific research with children's samples and data is crucial to gain further insights, particularly on the relationship between genes and the environment for genetic and multifactorial diseases.

Biobanks are well-placed to provide appropriate access to biological samples and data for pediatric research, but our review has identified a lack of inclusion and promotion of pediatric biobanks, notably at a European level, and a paucity of published research utilizing existing pediatric samples, including those from neonatal screening programmes. This led to a search for the specific issues that challenge pediatric biobanking.

We encountered the challenge of obtaining information as to where pediatric samples are available for research, even when using the BBMRI Directory/Negotiator. Then we explored specific ethical, legal, and societal issues that must be faced by researchers, research participants and citizens, with the support of legislation and soft law to ensure responsible research.

As we have already highlighted, children are a vulnerable population, and they are in need of adequate protective measures when they are involved in such research (5). Biobanks should aim to develop “a framework that provides a fair balance between fundamental pediatric research, privacy protection” (71) and special rights such as the right to an open future. Other specific issues relate to ensuring the engagement of participants in line with developing maturity, best guaranteed by a model of dynamic participatory “assent” with the minor participant and of permission by the parents/guardians.

Consequently, the current work highlights first the vulnerability of children followed by the five areas highlighted in the literature as critical in terms of ELS aspects in pediatric biobanks. These are: (i) the temporary vulnerability in children; (ii) the need for a risks and benefits assessment, including the right to an open future and return of secondary findings; (iii) legal majority; (iv) public awareness and empowerment and (v) challenges for research teams.

It is becoming increasingly clear that the current BBMRI-ERIC ELSI Knowledge Base needs to be strengthened to totally address these challenges. Additional steps need to be taken to enhance transparency in operational implementation of legal frameworks and best practice guidelines in pediatric research and more specifically in pediatric biobanking.

Moreover, as pediatric biobank research grows, we need an ethically sound balance between the protection of vulnerable populations and the development of FAIR (Findable, Accessible, Interoperable, and Reusable) research in the age of genomic medicine. It is thus critical to support researchers in rethinking the assent and consent process with minors, stressing that older children and adolescents are often developmentally capable of meaningful deliberation about the risks and benefits of participation in research. Conclusively, it is both an ELSI duty as well as a timely cultural investment to promote the children's capabilities and equip them (and indirectly the adults—their parents or guardians and their teachers), to recognize research biobanking as a pillar of scientific development and precision medicine. Biobanking can be understood as a practical platform for empowerment and engagement; without having to expose the participant to possible clinical risks, it provides understanding of the biomolecular turn of research and medicine, the impact of genetic knowledge on the life of each individual, on society in general, and the collaborative horizon at stake.

A number of reasons drove us to compose the current paper. These include the lack of findability of pediatric biobanks in databases, awareness of limitations in the current legislation and practical guidelines for pediatric biobanking, which are primarily directed toward clinical trials; the knowledge that the European Medical Association (EMA) is in the process of reviewing pediatric clinical trials (132); and the current extensive discussion on pediatric research (132, 133) and its ethical, legal and societal issues. As such this work is both timely as well as it lays the foundation for a more extensive future body of work on pediatric biobanking.

The review highlights how the role of the child in research, especially in biobanking, has changed dramatically in recent decades, due to the shift from a paternalistic approach to a fully participatory approach promoting the engagement of children in research as early as possible. Over time this paradigm shift has permeated our regulatory frameworks as well. It has been a long journey of inclusion and recognition of the child as a participant, whose opinion must be taken into account and whose dissent must be respected. At the same time, the current work outlines the path of making researchers and biobankers aware of their own role in potentially increasing the risk of vulnerability and discrimination of children involved in translational research if not acting responsibly. It is thus critical for biobanks to be an inclusive guarantor of empowerment, which can be achieved through capability building in different settings.

Additionally, biobanking practices themselves need to build on better defined training and educational activities, specifically targeted to pediatric biobanking as the direct translocation and implementation of practices from adult biobanking are simply inadequate. Crucially, pediatric biobanking needs to appropriately explain the potential long-term reach of research outcomes and provide a transparent governance framework to support those. Such actions and safeguards will expectantly empower all actors, and especially those most likely to benefit from providing their samples for research, the children themselves.

In conclusion, involving children at an early stage of research development ensures that projects are best suited to the children and that they “are not mere passive subjects but rather active participants in a joint enterprise of research,” that is “clinical research must thus always be *with* children and young people, not *on* them” (134).

## Author contributions

SC, BE, and MM contributed to the conception and design of the study. SC and BE wrote the first draft of the manuscript. EC was responsible for the primary literature search. All the authors contributed to review and editing of the manuscript and have read and approved the submitted version.

## Conflict of interest

The authors declare that the research was conducted in the absence of any commercial or financial relationships that could be construed as a potential conflict of interest.

## Publisher's note

All claims expressed in this article are solely those of the authors and do not necessarily represent those of their affiliated organizations, or those of the publisher, the editors and the reviewers. Any product that may be evaluated in this article, or claim that may be made by its manufacturer, is not guaranteed or endorsed by the publisher.

## IARC disclaimer

Where authors are identified as personnel of the International Agency for Research on Cancer/World Health Organization (IARC/WHO), the authors alone are responsible for the views expressed in this article and they do not necessarily represent the decisions, policy or views of the International Agency for Research on Cancer/World Health Organization.

## BBMRI-ERIC disclaimer

Where authors are identified as personnel of the Biobanking and BioMolecular resources Research Infrastructure-European Research Infrastructure Consortium (BBMRI-ERIC), the authors alone are responsible for the views expressed in this article and they do not necessarily represent the decisions, policy or views of BBMRI-ERIC.

## Footnotes

1 NetCord, was an international foundation of cord blood banks, which merged with the World Marrow Donor Association (WMDA) in 2017. The Foundation for the Accreditation of Cellular Therapy (FACT), is a non-profit corporation co-founded by the International Society for Cell and Gene Therapy (ISCT) and the American Society for Transplantation and Cellular Therapy (ASTCT) for the purposes of voluntary inspection and accreditation in the field of cellular therapy. http://www.factwebsite.org/cbstandards/ (accessed September 19, 2020).
